# Digital Patient-Controlled Follow-Up in Palliative Cancer Care: Protocol for a Mixed Methods Feasibility Study

**DOI:** 10.2196/92046

**Published:** 2026-08-11

**Authors:** Erik Torbjørn Løhre, Morten Thronæs, Hilde Hjelmeland Ahmedzai, Guro Birgitte Stene, May Aasebø Hauken, Line Merethe Oldervoll

**Affiliations:** 1 Centre for Crisis Psychology Faculty of Psychology University of Bergen Bergen Norway; 2 Department of Clinical and Molecular Medicine Faculty of Medicine and Health Sciences Norwegian University of Science and Technology (NTNU) Trondheim Norway; 3 Cancer Clinic St. Olavs University Hospital Trondheim Norway; 4 Department of Neuromedicine and Movement Science Faculty of Medicine and Health Science Norwegian University of Science and Technology (NTNU) Trondheim Norway

**Keywords:** palliative care, oncology, cancer, digitalization, digital health intervention, DHI, follow-up

## Abstract

**Background:**

Patient-centered care, emphasizing autonomy and shared decision-making, is essential in palliative cancer care. The increasing prevalence of cancer requires optimized health care use, and given patients’ preference for home-based care, traditional time-based follow-up appointments may not adequately address their needs. We have developed a digital patient-controlled follow-up intervention at the acute palliative care unit in Norway. The digital app is integrated into the existing national health service platform, MyHealth, and facilitates symptom monitoring, self-management support, and patient-controlled access to palliative care services.

**Objective:**

This pilot study will evaluate the feasibility and acceptability of the intervention and study procedures prior to examining the intervention’s effect in a future randomized controlled trial.

**Methods:**

Applying a mixed methods design, we plan to recruit 20 patients to participate in a 6-week intervention period, during which they will complete weekly electronic versions of the revised Edmonton Symptom Assessment System to monitor symptom burden and enable tailored follow-up. Participants will have access to a dedicated website providing self-management guidance and will also be able to electronically book appointments with the palliative care team. Health-related quality of life will be assessed at baseline and after the intervention using the European Organisation for Research and Treatment of Cancer Quality of Life Questionnaire C-15-Palliative. Following the intervention, patients will complete a satisfaction questionnaire. Semistructured interviews with patients and family carers will explore experiences, benefits, and barriers, while a focus group with health care professionals will assess feasibility from a clinical perspective. Quantitative and qualitative data will be analyzed separately before being integrated to identify convergence, complementarity, or divergence.

**Results:**

Findings from this pilot study will inform the design and feasibility of a future RCT. The recruitment of patients will start in October 2026, and the recruitment period and the intervention will last until April 2027 or until 20 patients have been recruited. We subsequently anticipate completing data analyses, manuscript writing, and submission of the stage 2 manuscript by October 2027. In January 2026, we received funding for the 4-year project (including a randomized controlled trial). This project is funded by the Dam Foundation (grant research [2025] #SDAM_FOR701622), supported by the Norwegian Cancer Society.

**Conclusions:**

Digital, need-based follow-up may offer a sustainable, patient-centered model for palliative cancer care. The findings of this feasibility study will inform the design of a randomized controlled trial to examine the effect of this intervention.

**International Registered Report Identifier (IRRID):**

PRR1-10.2196/92046

## Introduction

Cancer prevalence and the number of patients living with metastatic cancer are increasing [1,2]. Despite improved treatment options, a large proportion of patients with cancer are treated with noncurative intent [3]. Patients with advanced cancer receiving tumor-directed therapies often experience bothersome symptoms that benefit from the integration of oncological and palliative care services [4,5]. For patients with cancer receiving palliative care who are not receiving disease-modifying treatment, the symptom burden is high and increasing [4,6]. Additionally, these patients regularly face psychosocial challenges and spiritual distress [7,8]. These factors underscore the need for frequent contact with the health care system. However, patients’ preference to spend as much time as possible at home may appear to conflict with this need [5].

Patient-centered care and autonomy are acknowledged focal points in palliative care [5]. Shared decision-making is a key attribute of person-centered care, meaning that the patient perspective regarding treatment and follow-up choices is important [9]. Thus, in a landscape of increasing prevalence of metastatic cancer, limited available resources, and overuse of medical services, appropriate use of health care funding while maintaining focus on the patients’ point of view is crucial [10].

Although patients receiving palliative care traditionally are followed according to time-based preplanned visits, a substantial number of patients need unscheduled appointments [11]. Previous research has indicated that a need-based and personalized process for accessing palliative care services is relevant throughout the trajectory [11-13]. Still, even in the era of digital health interventions in palliative care, a recent systematic review included mostly studies that investigated the effects of time-based follow-up programs [14]. Given the extensive number of patients living with noncurative cancer, combined with a limited number of available health care professionals (HCPs), current models may not be sustainable [15]. Patient-initiated follow-up models are proposed as potential solutions, and supported self-management programs may even improve clinical, psychosocial, and economic outcomes [15]. Additionally, contacts with the health care system based solely on the patient’s need may increase patient autonomy and reduce time toxicity, a concept that describes the significant amount of time patients spend attending appointments that disrupt daily life [16]. For patients with advanced cancer, for whom time is especially precious, this may be particularly relevant [17].

The ongoing digital transformation in health care represents a step toward a more equitable service, and digital health interventions may be applicable in palliative care [14,18,19]. However, several potential barriers and concerns from the patient’s viewpoint have been identified [20]. These range from technical and operational issues to communication challenges and concerns about having an insufficient basis for making clinically sound decisions [20]. In addition, palliative care aims to support the family of the patients as well [21]. This implies that intended service alterations should also be evaluated with respect to their impact on family caregivers. Digital and on-demand follow-up may address multiple concerns and preserve both the patient’s perspective and support for families. For patients receiving tumor-directed treatment, hospital visits are usually required, but for patients receiving palliative care only, digital, patient-controlled follow-up may enable patients with cancer in outpatient palliative care settings to stay in their homes for longer periods while maintaining adequate symptom control and satisfactory quality of life (QoL). Nevertheless, measuring the effects of multifaceted interventions in complex health care systems is challenging [22]. A recommended first step is to investigate the feasibility of a planned intervention, in accordance with accepted criteria for study design [23,24].

We have developed a digital follow-up intervention comprising electronic symptom monitoring, self-management support, and patient-controlled access to care. In preparation for a randomized controlled trial (RCT), we will conduct a 6-week pilot study to investigate the feasibility of the intervention and study procedures. The research questions are as follows: (1) How do patients with cancer receiving palliative care who are not receiving ongoing oncological follow-up experience digital follow-up? (2) What do patients and carers report as the perceived benefits and barriers of the intervention? (3) What is the feasibility of recruiting and retaining study participants? (4) Are the patients able to comply with the study procedures, including completing digital questionnaires? (5) What is the use of participants’ hospital health care services during the intervention period?

## Methods

### Research Design

This is a prospective pilot study exploring the feasibility of digital patient-controlled access to palliative cancer care. It is underpinned by the Framework for Developing and Evaluating Complex Interventions [25] and addresses the research questions with a mixed methods approach [26]. The study’s quantitative strand is based on a single-center, single-arm, pretest and posttest design, and the qualitative strand is based on a descriptive research design. The data will be collected concomitantly from the same sample and analyzed separately before being ultimately combined to broaden the understanding of feasibility aspects [27].

### Setting

The study will be conducted at the acute palliative care unit (APCU), Cancer Clinic, St. Olavs Hospital, which is a European Society for Medical Oncology (ESMO)–designated center of integrated oncology and palliative care. The hospital has an electronic medical record system, with the integrated MyHealth app allowing mutual communication between health care providers and patients. All patients in the Mid-Norway Health Authority, where St. Olavs Hospital, Trondheim University Hospital, is situated, have access to MyHealth. The app is available in Norwegian only, although additions in other languages are under development.

The APCU consists of an outpatient clinic and a hospital ward located on the same floor. The outpatient clinic conducts approximately 1500 consultations each year. It also serves family physicians and home care services, allows direct contact with patients and relatives, and conducts weekly home visits. The hospital ward has 12 single-bed rooms, about 550 annual admissions, and an average hospital length of stay of 1 week. The unit is staffed with 36 nurses and 7 physicians, of whom 5 are senior oncologists with competency in specialized palliative care and 2 are junior physicians. Additionally, a multidisciplinary team consisting of physiotherapists, chaplains, an occupational therapist, a clinical dietitian, and a social worker is available for both patients and their family members.

Patients with cancer receiving palliative care, regardless of whether they are receiving oncological treatment, are referred to the unit. Patients with lung, gynecologic, and hematological malignancies are cared for by their respective hospital departments. As part of standard care, patients referred to the outpatient clinic are initially contacted by telephone by a designated nurse for information and triage. This is followed by an appointment with a palliative care physician and a nurse. Thereafter, consecutive visits are planned. Patients receiving tumor-directed treatment are followed by both an oncologist responsible for the cancer treatment and a palliative care physician, separately or in joint consultations, while patients receiving palliative care only are followed by a palliative care physician.

### Participants

We aim to recruit 20 adults with incurable cancer. In the context of a feasibility study, this number is methodologically appropriate and ethically justified [23,28]. No sample size calculations are provided due to the pilot nature of this study. Feasibility studies aim to assess practical aspects, such as recruitment, retention, data collection procedures, and acceptability of the intervention, rather than test hypotheses or estimate treatment effects. According to published guidelines, sample sizes between 12 and 30 participants are considered sufficient to evaluate feasibility outcomes and inform the design of future trials [28]. In the context of palliative care, where patients often experience a high symptom burden and have limited time, a smaller sample minimizes participant burden while still providing meaningful insights into feasibility, compliance, and preliminary outcome trends. This approach ensures ethical sensitivity and aligns with best practices for feasibility research in vulnerable populations.

All patients referred to the APCU will be screened for eligibility at their first or second appointment at the outpatient clinic by 1 of the 2 palliative care physicians involved in the study. Eligible patients will be given brief information about the study and asked if they are willing to receive more information about it. For patients willing to receive further information, the palliative care physician will contact the research nurse, who will come to the outpatient clinic to provide study information and potentially obtain written informed consent. We will also invite the primary carer of all included patients to participate in a postintervention patient-carer dyadic interview. They will be given a separate information letter by the research nurse, who will also obtain consent from those willing to participate in the interview.

Furthermore, all HCPs involved in recruiting patients will be invited to participate in a focus group interview at the end of the study. The rationale for this is to explore the experiences of carers and HCPs to further inform the feasibility and acceptability of the digital on-demand intervention. We anticipate conducting 1 focus group with 6 to 8 participants. Eligibility criteria for study participants are outlined in the next section.

### Eligibility Criteria

Inclusion criteria for patients are as follows: (1) being referred to the APCU, (2) being diagnosed with locally advanced or metastatic cancer, (3) not receiving oncological follow-up, and (4) being an adult (aged ≥18 years). Exclusion criteria include (1) patients with lung, gynecologic, or hematological malignancies under follow-up by other departments; (2) cognitive impairment preventing informed consent or participation; (3) language barriers preventing study compliance; and (4) currently receiving oncological follow-up.

Inclusion criteria for carers are as follows: (1) being the primary caregiver of a patient included in the study and (2) being willing and able to provide informed consent and participate in the study. Exclusion criteria include (1) medical or cognitive impairment preventing participation and (2) language barriers.

Inclusion criteria for HCPs are as follows: (1) working at the APCU outpatient clinic, (2) being trained in the intervention, and (3) being involved in the recruitment and follow-up of study participants.

### Intervention

The overall goal of the intervention is to provide safe follow-up with preserved patient autonomy and minimal time toxicity. Specifically, the intervention is a digital follow-up app that has been incorporated into MyHealth, which is an integrated part of the hospital’s electronic medical record system. The app facilitates mutual communication between health care providers and patients. The intervention aligns with the Norwegian health care authorities’ strategy for cancer care [29], emphasizing patient-controlled digital access to palliative care follow-up. It is a multilayered intervention, serving 3 purposes.

The first purpose is symptom monitoring. Once weekly, patients will complete an electronic version of the revised Edmonton Symptom Assessment System (ESAS-r) questionnaire [30]. Data will be submitted electronically to the APCU outpatient clinic via MyHealth, enabling HCPs to monitor patients’ symptoms and contact them in case of symptom worsening.

The second purpose is self-management support. Patients will gain access to a designated website offering guidance on symptom self-management and care in general, thus promoting patient autonomy and informed decision-making.

The third purpose is patient-controlled access to care. Patients and family carers can electronically book appointments or request contact with the APCU at a time and setting of their choice. This will ensure access to health care services when needed rather than through prearranged outpatient clinic appointments. Participants will be asked to report their need for contact on a weekly basis. In addition, consistent with current standard procedures, patients and family carers will be encouraged to contact the APCU at any time outside the scheduled weekly assessments if required, using the provided telephone number. Similarly, should the digital system fail or patients experience problems using it, they should contact the APCU immediately.

Health care personnel will monitor messages from patients daily; this applies to weekends and holidays as well. In the event of newly developed high symptom burden for which daily digital follow-up is insufficient, patients or family carers should contact the department directly by telephone. During regular working hours, this ensures immediate contact with a study physician; outside these hours—or at any other time when study physicians are unavailable for any reason—patients will get a consultation with the on-call physician at the cancer clinic. The situation will be managed as an emergency event, following the same procedures used in standard care at the department.

Likewise, HCPs at the APCU may contact patients at any time when medically indicated. Hence, the intervention will offer patients safe follow-up without unnecessary time away from their homes. Patients consenting to the study will be trained in using the intervention components in the MyHealth digital app and invited to use them for 6 weeks.

Criteria for discontinuing the intervention will be admission to the hospital for the remaining time of the intervention or a participant’s unwillingness to continue.

### Public Involvement

The study was designed in collaboration with, and with input from, the cancer clinic’s user panel for research. A designated user representative, appointed by the Norwegian Cancer Society, played an important part in the development of the intervention and will do so during the remaining work. Specifically, the user representative provided feedback on the concept of the intervention when it was first presented to the user involvement panel. They further provided input on the patient information letter and the protocol for the study. Furthermore, during June 2026 and July 2026, a total of 2 user representatives were involved in prepilot testing of the digital app. They will also have an important role in discussing the results from the feasibility study and planning the next steps in the research.

### Assessments and Procedures

#### Quantitative Data Collection

Demographic and medical data will be obtained from the participants’ electronic medical records. Symptom burden will be assessed weekly by using the validated self-report questionnaire (ESAS-r) [30,31] (Table 1). Health-related QoL is intended as the primary outcome in the future RCT, and we will include the European Organisation for Research and Treatment of Cancer (EORTC) Quality of Life Questionnaire (QLQ) C-15-Palliative in this pilot study to assess its acceptability and suitability as an outcome measure for the trial. This is a validated, abbreviated version of the EORTC QLQ-C30, specifically designed to assess health-related QoL in patients with advanced, incurable cancer receiving palliative care [32]. QoL will be assessed at baseline and at the end of the study (week 6). At this point, study participants will also complete an intervention-specific evaluation questionnaire assessing their level of satisfaction with the intervention. We will further collect data on recruitment rates, consent rates, attrition, reasons for not consenting, questionnaire completion rates, and use of the electronic appointment booking system and self-management program.

**Table 1:** Schedule of participant assessments.

Measures	Assessment types	Week 0	Week 1	Week 2	Week 3	Week 4	Week 5	Week 6
Demographic and medical data	Medical records	✓						
Electronic appointment booking	Patient-reported need for contact with the palliative care team		✓	✓	✓	✓	✓	✓
Symptom burden	Self-reported revised Edmonton Symptom Assessment System	✓	✓	✓	✓	✓	✓	✓
Quality of life	Self-reported European Organisation for Research and Treatment of Cancer Quality of Life Questionnaire C-15-Palliative	✓						✓
Intervention evaluation	Intervention-specific questionnaire							✓
Intervention evaluation and participant research burden	Semistructured interview with patients and primary carers							✓

#### Qualitative Data Collection

Qualitative data will be collected from semistructured interviews with patients and primary family carers and from a focus group interview with HCPs involved in the intervention. The interviews will be conducted at the end of the 6-week intervention period by researchers with expertise in qualitative methods and palliative care, who have not participated in recruitment or intervention delivery.

All study participants and their primary carers will be invited to participate in a dyadic in-depth interview, conducted at a location convenient for them (eg, at home or at the APCU) at the end of the 6-week intervention period. A semistructured interview guide will explore participants’ experiences with both the digital intervention and participation in the study. The opening question will be: “Can you please tell me how you have experienced the digital follow-up intervention?” Participants will be encouraged to speak freely, with follow-up questions focusing on lived experiences; satisfaction with the intervention; perceived benefits and barriers; and potential unintended effects, such as delayed contact, digital failure, and emotional distress. We will further explore the impact on carers, including potential increased caregiver burden, and research participation.

The focus group interview with HCPs will be conducted at the APCU outpatient clinic. Applying a semistructured interview guide, the focus group will explore HCPs’ experiences with both the digital intervention and the research process. It will specifically focus on the feasibility and acceptability of the intervention and study procedures. In addition, the focus group discussion will inform the assessment of intervention fidelity and any modifications to the intervention during the study period.

All interviews and the focus group discussion will be digitally recorded and transcribed verbatim. To ensure anonymity, identifying information will be excluded from the transcripts, and participants will be assigned pseudonyms.

### Data Handling

Data submitted by participants through the MyHealth app will be stored in the app. This is part of the national health record system (the Health Platform) and is protected by national digital solutions for patient records. The Health Platform was developed by Epic Health Care Community [33] and is used worldwide. Participant data submitted for the study will have restricted access for HCPs involved in the study, that is, physicians and nurses at the palliative care unit and research nurses responsible for data collection. These restrictions will be created in the digital system. Data extracted for analysis will be stored in secure data solutions at the University of Bergen.

### Analyses

Quantitative and qualitative data will be analyzed independently before being merged for the final interpretation. The analysis plan focuses on feasibility outcomes, missing data patterns, and descriptive change scores.

#### Statistical Analysis

Baseline demographics will be summarized using descriptive statistics and reported with relevant measures, such as 95% CIs. Continuous variables will be summarized using means and SDs (or medians and IQRs if skewed). Categorical variables will be presented as frequencies and percentages. In addition, 95% CIs will be reported for key feasibility outcomes (Table 2).

Feasibility and acceptability of the patient-reported outcome measures (PROMs) will be assessed by calculating completion rates at all time points. Completion rates will be reported as percentages and frequencies. The distribution of data from the PROMs will be analyzed and presented as means with SDs. Missing data patterns and descriptive change scores will be reported. Quantitative data will be analyzed using SPSS (version 31.0; IBM Corp) software.

**Table 2:** Feasibility outcomes framework.

Feasibility domains, outcomes, and assessments				Progression threshold
**Demand**				
	**Study recruitment**			
		Number recruited within 6 months	≥70% of 20 patients	
		Proportion of eligible patients who consented to participate	≥70%	
		Reasons for refusals	N/A^a^	
	Health care use: extent of health care consultations			NPC^b^
**Acceptability**				
	**Intervention adherence and acceptability**			
		Retention rate	NPC	
		Reasons for dropout	N/A	
		Intervention compliance (use of intervention features, including digital reporting of symptoms and consultation needs)	≥70% of participants using the digital tool as intended	
		Intervention evaluation questionnaire	≥70% reporting “Quite satisfied” and “Very satisfied” overall	
		Patient and carer interviews	NPC	
	Impact on carers: patient and carer interviews			NPC
**Implementation**				
	**Intervention fidelity**			
		Focus group with health care professionals	NPC	
		Number and nature of intervention modifications	NPC	
	Technical aspects: focus group with health care professionals			NPC
**Practicality: assessment procedures and research burden**				
	Questionnaire completion rates			≥70%
	Patient and carer interviews			NPC
Responsiveness (quality of life): changes in quality of life measures				NPC

^a^N/A: not applicable.

^b^NPC: no progression criterion.

#### Analyses of Qualitative Data

Qualitative data will be analyzed using systematic text condensation (STC), a 4-step method well suited for thematic cross-case analysis [27]. The analyses will be performed by researchers skilled in qualitative research and analysis, and the process includes 4 steps.

First, initial reading and theme identification: researchers will independently read the transcripts and then meet to discuss preliminary themes. Second, coding and identification of meaning units: transcripts will be reread and coded individually, followed by consensus discussions to define meaning units. Coding will be supported by NVivo (Lumivero LLC) software. Third, condensation and categorization: meaning units will be grouped into categories and subcategories through collaborative analysis. Fourth, synthesis and validation: themes and subthemes will be validated against the original transcripts, with illustrative quotes extracted to support the findings.

STC is an iterative process, allowing movement between steps to refine interpretations. Reflexivity will be maintained throughout, with attention to context and researchers’ preconceptions. To ensure transparency, the analytic process will be outlined in a table, and findings will be validated against the transcripts to confirm that participants’ intended meanings are accurately represented.

#### Mixed Methods Integration and Analysis

In the final phase of the analyses, qualitative and quantitative findings will be merged to generate a comprehensive understanding of the intervention’s feasibility. This will be presented in a joint display to visually and narratively compare findings, enabling the identification of areas of convergence; complementarity; or divergence related to the feasibility factors, symptom levels, and QoL. Convergence indicates mutual reinforcement of findings, complementarity reflects added depth or nuance, and divergence highlights inconsistencies warranting further exploration. The merged results will be summarized and interpreted to inform the future RCT of the intervention [26].

### Criteria for Feasibility Success

We have selected 5 key outcomes for determining feasibility success and progression to a randomized controlled study. These relate to recruitment rates, consent rates, intervention compliance, intervention evaluation, and questionnaire completion rates. Progression criteria for the 5 key feasibility outcomes are presented in Table 2. Despite its importance in feasibility studies, we did not set a progression threshold for study retention due to an expected high level of attrition. We would, for instance, expect that some of the study participants may die during the project, although that would not necessarily indicate a lack of feasibility of the intervention. However, the retention rate will be analyzed and considered when estimating the power of a future RCT. The chosen threshold criteria were determined to be appropriate considering the anticipated deterioration of the population under investigation [34,35]. If feasibility outcomes do not meet the a priori–determined thresholds, an RCT may still be pursued if appropriate modifications can be made to the intervention or study procedures to support a successfully conducted RCT.

As displayed in Table 2, the study will investigate further feasibility outcomes—with no progression criteria—relating to the domains of demand, acceptability, implementation, practicality, and responsiveness. Although not pivotal for determining progression to an RCT, these outcomes will inform the design and planning of a potential future intervention trial.

### Ethical Considerations

The study will adhere to the Declaration of Helsinki and its amendments, the conventions of the Council of Europe on Human Rights and Biomedicine, and the corresponding Norwegian laws. All data will be stored on the institution’s secure research server in accordance with national and institution-specific regulations. The study has been approved by the South-East Norway Regional Committee for Medical Research Ethics on December 8, 2025 (938904). All patients will provide written informed consent before enrollment in the study. Written informed consent will be obtained by a research nurse. The study will be registered with the Open Science Framework following in-principle acceptance.

## Results

Findings from this pilot study will inform the design and feasibility of a future RCT. Results, including participant flow, will be presented in line with the CONSORT (Consolidated Standards of Reporting Trials) extension to pilot and feasibility trials [36]. This will offer insight into the suitability of digital follow-up interventions for this population as a whole and for subgroups such as those defined by age, educational level, stage of cancer, and marital status. It will further provide insight into changes in symptom burden, QoL, and health care use. Participant characteristics and feasibility outcome findings will be presented in a combination of text and tables.

The recruitment of patients will start in October 2026, and the recruitment period and the intervention will last until April 2027 or until 20 patients have been recruited. We subsequently anticipate completing data analyses, manuscript writing, and submission of the stage 2 manuscript by October 2027.

As of July 14, 2026, we received funding for the preparation of a stage 1 publication in May 2025, and funding for a 4-year project (including an RCT) was received in January 2026. This project is funded by the Dam Foundation (grant research [2025] #SDAM_FOR701622), supported by the Norwegian Cancer Society. These organizations had no role in the design or reporting of the protocol.

## Discussion

### Clinical Implications

Research on this topic has several potential implications for clinical practice in palliative cancer care. Conforming to the core principles of patient-centered care, the intervention supports shared decision-making, promoting patient autonomy. Weekly electronic symptom reporting allows timely identification of symptom deterioration and rapid clinical response. By replacing rigid, time-based follow-up schedules with need-based contact, the model may reduce unnecessary hospital visits and associated time burdens. For patients with advanced cancer, this could preserve valuable time and improve overall QoL. Including family carers in the intervention acknowledges their role in care and may impact caregiver burden. This holistic approach aligns with the goals of palliative care to support both patients and their families.

### Study Limitations

The study will comprise a small sample size and be conducted at a single center. However, this is considered appropriate for a feasibility study testing a new intervention, for which generalizability of the results is not applicable. The 6-week intervention period is short, and although it is sufficiently long to test the feasibility of the digital follow-up tool, it may not fully capture the outcome of patients’ health care use. Furthermore, patients with limited digital knowledge may introduce selection bias, potentially excluding some patients and limiting equity in care delivery. The extent of this bias will be determined from recruitment rates and reasons for nonconsent to study participation.

### Conclusions

This pilot study will provide critical insights into the feasibility and acceptability of a digital, patient-controlled follow-up model in palliative cancer care. If successful, the intervention could represent a paradigm shift toward more flexible, patient-centered care, reducing time toxicity and improving QoL. The findings will inform the design of a subsequent RCT and may contribute to national strategies for integrating digital health interventions into palliative care.

## Appendix

Multimedia Appendix 1 SPIRIT checklist.

## Abbreviations

APCU: acute palliative care unit
CONSORT: Consolidated Standards of Reporting Trials
EORTC: European Organisation for Research and Treatment of Cancer
ESAS-r: revised Edmonton Symptom Assessment System
ESMO: European Society for Medical Oncology
HCP: health care professional
PROM: patient-reported outcome measure
QLQ: Quality of Life Questionnaire
QoL: quality of life
RCT: randomized controlled trial
STC: systematic text condensation

## References

[1] De Angelis R, Demuru E, Baili P, Troussard X, Katalinic A, Chirlaque Lopez MD, Innos K, Santaquilani M, Blum M, Ventura L, Paapsi K, Galasso R, Guevara M, Randi G, Bettio M, Botta L, Guzzinati S, Dal Maso L, Rossi S (2024). Complete cancer prevalence in Europe in 2020 by disease duration and country (EUROCARE-6): a population-based study. Lancet Oncol.

[2] Gallicchio L, Devasia TP, Tonorezos E, Mollica MA, Mariotto A (2022). Estimation of the number of individuals living with metastatic cancer in the United States. J Natl Cancer Inst.

[3] Fekete Z, Fekete A, Kacsó G (2024). Treatment classification by intent in oncology-the need for meaningful definitions: curative, palliative and potentially life-prolonging. J Pers Med.

[4] Thronæs M, Løhre ET, Kvikstad A, Brenne E, Norvaag R, Aalberg KO, Moen MK, Jakobsen G, Klepstad P, Solberg A, Solheim TS (2021). Interventions and symptom relief in hospital palliative cancer care: results from a prospective longitudinal study. Support Care Cancer.

[5] Kaasa S, Loge JH, Aapro M, Albreht T, Anderson R, Bruera E, Brunelli C, Caraceni A, Cervantes A, Currow DC, Deliens L, Fallon M, Gómez-Batiste X, Grotmol KS, Hannon B, Haugen DF, Higginson IJ, Hjermstad MJ, Hui D, Jordan K, Kurita GP, Larkin PJ, Miccinesi G, Nauck F, Pribakovic R, Rodin G, Sjøgren P, Stone P, Zimmermann C, Lundeby T (2018). Integration of oncology and palliative care: a Lancet Oncology Commission. Lancet Oncol.

[6] Seow H, Barbera L, Sutradhar R, Howell D, Dudgeon D, Atzema C, Liu Y, Husain A, Sussman J, Earle C (2011). Trajectory of performance status and symptom scores for patients with cancer during the last six months of life. J Clin Oncol.

[7] Sultana A, Tasnim S, Sharma R, Pawar P, Bhattcharya S, Hossain MM (2021). Psychosocial challenges in palliative care: bridging the gaps using digital health. Indian J Palliat Care.

[8] Gijsberts MJ, Liefbroer AI, Otten R, Olsman E (2019). Spiritual care in palliative care: a systematic review of the recent European literature. Med Sci (Basel).

[9] Baik D, Cho H, Masterson Creber RM (2019). Examining interventions designed to support shared decision making and subsequent patient outcomes in palliative care: a systematic review of the literature. Am J Hosp Palliat Care.

[10] Brownlee S, Chalkidou K, Doust J, Elshaug AG, Glasziou P, Heath I, Nagpal S, Saini V, Srivastava D, Chalmers K, Korenstein D (2017). Evidence for overuse of medical services around the world. Lancet.

[11] Azhar A, Wong AN, Cerana AA, Balankari VR, Adabala M, Liu DD, Williams JL, Bruera E (2018). Characteristics of unscheduled and scheduled outpatient palliative care clinic patients at a comprehensive cancer center. J Pain Symptom Manage.

[12] Hui D, Bruera E (2020). Models of palliative care delivery for patients with cancer. J Clin Oncol.

[13] Hui D, Heung Y, Bruera E (2022). Timely palliative care: personalizing the process of referral. Cancers (Basel).

[14] Zhao B, Zhang S, Zhang T, Chen Y, Zhang C (2024). Effect of applying digital health in palliative care for patients with advanced cancer: a meta-analysis and systematic review. Support Care Cancer.

[15] Dretzke J, Lorenc A, Adriano A, Herd C, Mehanna H, Nankivell P, Moore DJ (2023). Systematic review of patients' and healthcare professionals' views on patient-initiated follow-up in treated cancer patients. Cancer Med.

[16] Nwozichi C, Omolabake S, Ojewale MO, Faremi F, Brotobor D, Olaogun E, Oshodi-Bakare M, Martins-Akinlose O (2024). Time toxicity in cancer care: a concept analysis using Walker and Avant's method. Asia Pac J Oncol Nurs.

[17] Gupta A, Brundage MD, Galica J, Karim S, Koven R, Ng TL, O'Donnell J, tenHove J, Robinson A, Booth CM (2024). Patients' considerations of time toxicity when assessing cancer treatments with marginal benefit. Oncologist.

[18] Stoumpos AI, Kitsios F, Talias MA (2023). Digital transformation in healthcare: technology acceptance and its applications. Int J Environ Res Public Health.

[19] Finucane AM, O'Donnell H, Lugton J, Gibson-Watt T, Swenson C, Pagliari C (2021). Digital health interventions in palliative care: a systematic meta-review. NPJ Digit Med.

[20] Kirby A, Griffin D, Heavin C, Drummond FJ, McGrath C, Kiely F (2025). Telehealth adoption in palliative care: a systematic review of patient barriers and facilitators. BMC Palliat Care.

[21] The Lancet Regional Health-Americas (2024). Dignity and support after cancer: we need to talk about palliative care. Lancet Reg Health Am.

[22] Braithwaite J (2018). Changing how we think about healthcare improvement. BMJ.

[23] Teresi JA, Yu X, Stewart AL, Hays RD (2022). Guidelines for designing and evaluating feasibility pilot studies. Med Care.

[24] Bowen DJ, Kreuter M, Spring B, Cofta-Woerpel L, Linnan L, Weiner D, Bakken S, Kaplan CP, Squiers L, Fabrizio C, Fernandez M (2009). How we design feasibility studies. Am J Prev Med.

[25] Skivington K, Matthews L, Simpson SA, Craig P, Baird J, Blazeby JM, Boyd KA, Craig N, French DP, McIntosh E, Petticrew M, Rycroft-Malone J, White M, Moore L (2021). A new framework for developing and evaluating complex interventions: update of Medical Research Council guidance. BMJ.

[26] Creswell JW, Plano Clark VL (2017). Designing and Conducting Mixed Methods Research.

[27] Aschbrenner KA, Kruse G, Gallo JJ, Plano Clark VL (2022). Applying mixed methods to pilot feasibility studies to inform intervention trials. Pilot Feasibility Stud.

[28] Montgomery R (2025). Sample size justification in feasibility studies: moving beyond published guidance. Pilot Feasibility Stud.

[29] Joint efforts against cancer: national cancer strategy (2025–2035). Norwegian government.

[30] Watanabe SM, Nekolaichuk C, Beaumont C, Johnson L, Myers J, Strasser F (2011). A multicenter study comparing two numerical versions of the Edmonton Symptom Assessment System in palliative care patients. J Pain Symptom Manage.

[31] Hui D, Bruera E (2017). The Edmonton Symptom Assessment System 25 years later: past, present, and future developments. J Pain Symptom Manage.

[32] Groenvold M, Petersen MA, Aaronson NK, Arraras JI, Blazeby JM, Bottomley A, Fayers PM, de Graeff A, Hammerlid E, Kaasa S, Sprangers MA, Bjorner JB (2006). The development of the EORTC QLQ-C15-PAL: a shortened questionnaire for cancer patients in palliative care. Eur J Cancer.

[33] EPIC.

[34] Voruganti T, Grunfeld E, Jamieson T, Kurahashi AM, Lokuge B, Krzyzanowska MK, Mamdani M, Moineddin R, Husain A (2017). My team of care study: a pilot randomized controlled trial of a web-based communication tool for collaborative care in patients with advanced cancer. J Med Internet Res.

[35] Thabane L, Ma J, Chu R, Cheng J, Ismaila A, Rios LP, Robson R, Thabane M, Giangregorio L, Goldsmith CH (2010). A tutorial on pilot studies: the what, why and how. BMC Med Res Methodol.

[36] Eldridge SM, Chan CL, Campbell MJ, Bond CM, Hopewell S, Thabane L, Lancaster GA (2016). CONSORT 2010 statement: extension to randomised pilot and feasibility trials. BMJ.

